# Methane Emission in a Specific Riparian-Zone Sediment Decreased with Bioelectrochemical Manipulation and Corresponded to the Microbial Community Dynamics

**DOI:** 10.3389/fmicb.2015.01523

**Published:** 2016-01-11

**Authors:** Elliot S. Friedman, Lauren E. McPhillips, Jeffrey J. Werner, Angela C. Poole, Ruth E. Ley, M. Todd Walter, Largus T. Angenent

**Affiliations:** ^1^Department of Biological and Environmental Engineering, Cornell University, IthacaNY, USA; ^2^Department of Chemistry, State University of New York College at CortlandCortland, NY, USA; ^3^Department of Molecular Biology and Genetics, Cornell University, IthacaNY, USA

**Keywords:** microbial iron reduction, riparian zones, methanogenesis, microbial food web, bioelectrochemical systems, microbial electrochemistry

## Abstract

Dissimilatory metal-reducing bacteria are widespread in terrestrial ecosystems, especially in anaerobic soils and sediments. Thermodynamically, dissimilatory metal reduction is more favorable than sulfate reduction and methanogenesis but less favorable than denitrification and aerobic respiration. It is critical to understand the complex relationships, including the absence or presence of terminal electron acceptors, that govern microbial competition and coexistence in anaerobic soils and sediments, because subsurface microbial processes can effect greenhouse gas emissions from soils, possibly resulting in impacts at the global scale. Here, we elucidated the effect of an inexhaustible, ferrous-iron and humic-substance mimicking terminal electron acceptor by deploying potentiostatically poised electrodes in the sediment of a very specific stream riparian zone in Upstate New York state. At two sites within the same stream riparian zone during the course of 6 weeks in the spring of 2013, we measured CH_4_ and N_2_/N_2_O emissions from soil chambers containing either poised or unpoised electrodes, and we harvested biofilms from the electrodes to quantify microbial community dynamics. At the upstream site, which had a lower vegetation cover and highest soil temperatures, the poised electrodes inhibited CH_4_ emissions by ∼45% (when normalized to remove temporal effects). CH_4_ emissions were not significantly impacted at the downstream site. N_2_/N_2_O emissions were generally low at both sites and were not impacted by poised electrodes. We did not find a direct link between bioelectrochemical treatment and microbial community membership; however, we did find a correspondence between environment/function and microbial community dynamics.

## Introduction

Riparian zones, which are the areas of land adjacent to streams, are often hotspots for biogeochemical transformations (Vidon et al., 2010). These ecosystems provide valuable services by acting as buffers between land and stream to prevent nutrients and pollutants from entering aquatic environments. Within riparian zones, subsurface microbial communities play a major role in biogeochemical cycling, impacting carbon and nitrogen availability, and, by extension, plant productivity (Groffman et al., 1992; Burgin et al., 2010). Past work has examined denitrification processes in riparian zones, because these zones typically provide the last opportunity to mitigate excess nitrate in groundwater before it reaches the stream. Studies have found that the main regulators of denitrification (e.g., nitrate, anaerobic conditions, availability of other terminal electron acceptors and carbon sources) are spatially and temporally heterogeneous, which makes modeling these landscape-level processes particularly difficult (McClain et al., 2003; Groffman et al., 2009; Ranalli and Macalady, 2010; Vidon et al., 2010). Therefore, it is essential to have a full understanding of the underlying biogeochemistry to inform landscape level models, determine best management practices, and guide regulatory policies.

Bioelectrochemical systems (BESs) are engineered systems that utilize a biocatalyst, such as bacteria or enzymes, at electrodes. Microbial BESs capitalize on the ability of, for example, dissimilatory metal-reducing bacteria to respire with solid-state electrodes via extracellular electron transfer (Logan et al., 2006; Lovley, 2008; Franks and Nevin, 2010; Hamelers et al., 2010). These BESs produce power in microbial fuel cells (MFCs; He et al., 2005; Fornero et al., 2010a,b; Rosenbaum et al., 2011b); produce chemical products in microbial electrolysis cells (Rozendal et al., 2009; Villano et al., 2010; Cheng and Logan, 2011; Cusick et al., 2011; Rosenbaum et al., 2011a); remediate pollutants (Gregory and Lovley, 2005; Strycharz et al., 2010; Morris and Jin, 2012); sense environmental and chemical parameters (Chang et al., 2005; Kumlanghan et al., 2007); and produce logic gates in biocomputing devices (Li et al., 2011; TerAvest et al., 2011). In the environment, potentiostatically poised electrodes (i.e., electrodes held at a constant electrical potential using an electrical device called a potentiostat), can mimic iron(III)- and humic substance-compounds and act as the terminal electron acceptor for dissimilatory metal-reducing bacteria (Williams et al., 2010; Zhang et al., 2010; Friedman et al., 2012, 2013). The advantage of using an electrode instead of, for example, iron(III) is that the electrode can act as an inexhaustible terminal electron acceptor, which can be set precisely to the potential of interest without any other chemical interactions with the community. Thus, BESs can be a powerful tool for precise manipulation of environmental conditions for *in situ* experimentation. Here, we utilized a BES to study the interactions between microbial communities and the presence of iron(III) and humic substances as potential terminal electron acceptors under anaerobic conditions in a riparian zone.

Dissimilatory metal reduction has been shown to dominate a wide variety of anaerobic soils and sediments from the tropics to the poles (Weber et al., 2006; Dubinsky et al., 2010; Lipson et al., 2010, 2013; Keller and Takagi, 2013). Thermodynamically, iron- and manganese-reduction yield less energy than denitrification but more than sulfate reduction and methanogenesis (Bethke et al., 2011; Regnier et al., 2011). Therefore, in the presence of, for example, iron(III) [or electrodes mimicking iron[III](Friedman et al., 2012, 2013)] under anaerobic conditions, methane emissions should decrease. Indeed, at least one study has clearly observed that the presence of electrodes in a MFC decreased methane production (Ishii et al., 2008), although this was conducted in the laboratory and not in natural soil environments. Thermodynamic calculations are made under ideal conditions and do not take into account the ecological and physiological factors encountered *in situ*. For example, our recent work in Arctic peat soils has shown that bioelectrochemical manipulation designed to enrich for dissimilatory metal reduction actually increased methane emissions from soils. In this case, there was likely a bottleneck in the degradation of plant organic matter, which was widened by bioelectrochemical manipulation and stimulated the production of fermentation-like products (Friedman et al., 2013).

Modeling efforts are generally concerned with landscape-level function, such as pollutant removal or greenhouse gas emissions, but the parameters used in the model are dependent on the microbial-scale processes within the ecosystem and can vary by several orders of magnitude (Schmidt et al., 2011; Palmer and Febria, 2012; Wood et al., 2012). Therefore, it is critical to elucidate the links between microbial community structure and ecosystem processes (Raes and Bork, 2008; Morales and Holben, 2011). Here, we focused on the microbial scale within a riparian zone and we combined *in situ* bioelectrochemical manipulation with quantitative measurements of community structure (assessed by 16S rRNA gene sequences), environmental parameters (i.e., soil temperature, pH), and ecosystem function (rates of CH_4_ and N_2_/N_2_O emissions). The objective of this work is to provide a foundation for elucidating the complex relationships between microbial community structure, community function, and ecosystem function (i.e., biogeochemical cycles); ultimately, the goal is to better inform modeling efforts through a more complete understanding of these complex relationships. One of our main objectives was to find correlations between changes of the environment, the community structure, and microbial function during the experimental period for several different experimental systems at two sites, rather than to describe the microbial community (α diversity) as a snapshot measurement, which does not inform about its function (Prosser, 2015).

At two distinct sites adjacent to an agricultural field within the riparian zone of Fall Creek in Freeville, NY, we measured CH_4_ and N_2_/N_2_O emissions from soil chambers with either potentiostatically poised electrodes (mimicking inexhaustible iron[III] or humic substances) or unpoised electrodes (control) during a sampling period of 6 weeks in the spring of 2013. We also gathered environmental data during the experiment, including: soil temperature, pH, dissolved oxygen concentration, conductivity, iron concentrations and speciation, and anion concentrations. Once per week, bacterial biofilms were harvested from both poised and unpoised electrodes for 16S rRNA gene sequencing to determine microbial community structure. Here, we demonstrated the capability of small alterations to redox conditions to impact carbon release to the atmosphere, and provide a foundation for future examinations of biogeochemical cycling using *in situ* bioelectrochemical manipulations. Our hypothesis was that placement of iron(III)/humic substance-mimicking electrodes would reduce the emissions of methane in a very specific riparian zone of Fall Creek in Freeville, NY, USA.

## Materials and Methods

### Field Location and Experimental Setup

This experiment was conducted within Fall Creek, which is a third order stream in Central New York that passes through the Homer C. Thompson Vegetable Research Farm and a Cornell University facility in Freeville, NY (42°31′N, 76°20′W; McPhillips et al., 2015). Six soil chambers were installed at each of two sites that were separated by ∼50 m. These sites are further denoted in the text as “upstream” or “downstream.” At each site, three of the chambers contained potentiostatically poised graphite electrodes (experimental), while the other three contained unpoised graphite electrodes (control). We installed both soil chambers and electrodes by first making small incisions in the sediment with a serrated knife and then by inserting the chambers/electrodes. We performed the experiment from April 25th through June 6th, 2013.

### Soil Chamber Construction

Soil chambers were constructed from 3.79 L plastic buckets (#2860, U.S. Plastic Corp., Lima, OH, USA). To create the base of the chamber, the bottom 2-cm of the bucket was removed leaving a 17-cm-long cylinder; when placed in the soil, 11-cm of the soil chamber was below the surface and 6-cm extended above the soil surface (Supplementary Figures S1–S3). From each chamber, an 8.5-cm × 4.5-cm section of the plastic material that would be in the subsurface area was removed and replaced with an anion exchange membrane (AMI-7001S, Membranes International, Glen Rock, NJ). To create a cap for the soil chamber, the top 4-cm was removed from another 3.79 L plastic bucket. The bottom edge of the plastic bucket was reinforced with foam insulation tape and duct tape to ensure an airtight seal between the soil chamber and the chamber cap (the cap was only used during short measurement periods). Two 0.635-cm cylindrical septa (AT6526, Fisher Scientific, USA) were affixed to the top of the chamber cap and sealed with urethane adhesive. During measurements, the chamber was vented with a 21G needle (#305129, VWR, USA) through one of the septa to prevent induced pressure differentials in the soil chamber (Hutchinson and Mosier, 1981). We took gas samples through the other septum.

### Bioelectrochemical Systems

The working and counter electrodes (CE) were manufactured from medium-extruded graphite plates (GT001135, Graphite Store, Buffalo Grove, IL, USA). The CE consisted of a 6-cm × 6-cm of 0.635-cm thick block (surface area = 87.24 cm^2^), while each working electrode (WE) consisted of six 6-cm × 6-cm × 0.635-cm blocks connected in parallel (total surface area = 523.44 cm^2^). Having multiple electrodes in parallel for the WEs allowed for the harvesting of biofilms during different stages of the experiment. The WE and reference electrode (RE) were placed inside the chamber, while the CE was placed outside the chamber on the opposite side of the membrane to maintain an electrical connection while preventing cathodic hydrogen or methane production inside the soil chamber. Electrodes were connected to microcontroller-based potentiostats (Friedman et al., 2012) by inserting the exposed end of a 3-m length of 18-gage stranded copper wire into a 1.59-mm hole drilled in the top of each graphite block. A conductive carbon adhesive (#12664, Electron Microscopy Sciences, Hatfield, PA, USA) was used to ensure a good electrical connection (resistance < 0.5 Ω), and the junction was sealed with a urethane adhesive (#4024, Hardman, South Bend, IN, USA). The WEs in experimental chambers were poised at 0.1 V_SHE_ using a microcontroller-based potentiostat (Friedman et al., 2012) and an Ag/AgCl RE was made in house. Electrodes in unpoised chambers were left at open circuit. Current data fluctuated throughout the experimental period due to many different factors, including diurnal changes (Supplementary Figures S4 and S5), and our data was not corrected for background chemical currents, chemical interactions, noise from electric fields, and open circuit potential for the unpoised electrodes.

### Measurements

Measurements were taken every Monday and Thursday during the 6-week experimental period. We measured denitrification rates using the acetylene inhibition method, where acetylene is added to the soil to inhibit reduction of N_2_O to N_2_ (Freney et al., 1992; Thompson, 1996; Groffman et al., 2006), allowing quantification of N_2_ and N_2_O emissions through the measurement of N_2_O only. Even though this method evaluates the denitrification rates (N_2_ production), we indicate this in the text as N_2_/N_2_O emissions. We prepared and used beeswax-coated calcium carbide tablets, which react with water to form acetylene gas, as described by Thompson (1996). Six beeswax-coated calcium carbide tablets were inserted into each soil collar 45 min prior to gas sampling at a depth of 7–15 cm. We measured acetylene concentrations in the gas samples to ensure that ample acetylene (>1% v/v) was being produced to inhibit N_2_O reduction (Thompson, 1996). Gas emissions were measured from the chambers by placing the cap on the chamber and collecting 12 mL of the headspace gas at four time points (0, 10, 20, and 30 min). Gas chromatography analysis of N_2_O, CH_4_, and C_2_H_2_ was performed on an Agilent 6890N gas chromatograph equipped with a HP 7694 Headspace Autosampler (Hewlett-Packard Company, Palo Alto, CA, USA). N_2_O separation was performed using a Supel-Q^TM^ PLOT capillary column (30 m × 0.32 mm; Supelco Inc., Bellafonte, PA, USA) with ultra-pure helium carrier gas (2.6 mL min^-1^) and 95:5 Ar:CH_4_ make-up gas (8.2 mL min^-1^) and an electron capture detector set to 250°C. CH_4_ and C_2_H_2_ separation was performed using a Carboxen 1006 PLOT capillary column (30 m × 0.32 mm; Supelco, Inc.) and a flame ionization detector set to 200°C with H_2_ gas (30 mL min^-1^), air (400 mL min^-1^), and N_2_ makeup gas (25 mL min^-1^). The oven temperature was initially set to -22°C for 4.7 min, then increased to 30°C for 0.85 min and finally increased to 80°C for 2.5 min to allow for elution of all three gasses of interest. Calibration curves were made using serial dilutions of 1 ppm N_2_O, 20 ppm CH_4_, and 2.5% C_2_H_2_ (Airgas Inc.). Emissions were calculated as the slopes of the linear regression (concentration vs. time) curves (*R*^2^ > 0.9) for each measurement period (flux).

Soil temperature, pH, dissolved oxygen, and conductivity were measured at 7-cm depth directly adjacent to each soil chamber using a portable multiparameter meter (Orion Star A329, Thermo Scientific, Pittsburgh PA, USA) and probes (ROSS Ultra Triode pH/ATC electrode, DuraProbe conductivity probe, Orion RDO probe, Thermo Scientific, Pittsburgh, PA, USA). Soil water was collected from within each soil chamber (2–7 cm below the soil surface) using porous soil moisture samplers (#220300, Rhizosphere, Wageningen, The Netherlands), vacutainers (VT6430, BD, Franklin Lakes, NJ, USA), and 21G needles (#305129, VWR, USA). These samples were analyzed for nitrate, nitrite, chloride, and sulfate concentrations using a Dionex ICS-2000 ion chromatograph with IonPac AS-18 analytical column and 25-μL sample loop. Immediately following the collection of soil water, 0.5 mL of each sample was transferred to another vacutainer containing 0.5 mL of 0.5 N HCl; these samples were analyzed for Fe^2+^ and total Fe using the ferrozine assay (Braunschweig et al., 2012).

### Microbial Community Analysis

Biofilms from the WEs of both experimental and control soil chambers were collected weekly throughout the 6-week period of the experiment. Each of the six parallel WEs in every soil chamber was harvested once during the course of the experiment to obtain a time series of the microbial community. We harvested biofilms by removing the electrode from the soil and by scraping the biofilm into a 15-mL sterile centrifuge tube (#93000-026, VWR, USA) using a sterile blade. Samples were placed on wet ice immediately in the field, and then stored at -20°C until the completion of the experiment. Following biofilm harvesting, the bare electrodes were returned to the soil to keep the WE surface area constant throughout the experiment. Genomic DNA was extracted using a PowerSoil^®^ DNA Isolation Kit (MoBio, Carlsbad, CA, USA). Bacterial extraction product was then amplified in duplicate 50-μL polymerase chain reactions (PCRs) according to Gilbert et al. (2010) using 25 cycles. In short, duplicate 50-μL PCR reactions were conducted using: 28 μL molecular grade water, 20 μL mastermix (5Prime Hot MasterMix, Catalog # 2200110, 5Prime, Fischer Scientific, USA), 0.5 μL 515f forward primer (Caporaso et al., 2012), 0.5 μL 806r barcoded reverse primer (Caporaso et al., 2012), and 1 μL template DNA. Reactions were run under the following conditions: 94°C for 3 min; then 94°C for 45 s, 50°C for 60 s, and 72°C for 90 s (repeat 25 times); 72°C for 10 min, and then hold at 4°C. Duplicate PCR products were then pooled, the presence of amplicons confirmed by gel electrophoresis, and products cleaned using the Mag-Bind^®^ E-Z Pure Kit (Omega, Norcross, GA, USA). Cleaned product was again confirmed with gel electrophoresis and barcoded amplicons for each sample were pooled at equimolar ratios to a final concentration of 8 ng DNA μL^-1^. The single, pooled amplicon mixture was sequenced at the Cornell University Biotechnology Resource Center using an Illumina MiSeq (2 × 250 bp, paired end). Data were quality filtered with the quantitative insights into microbial ecology (QIIME) platform (v 1.6; Caporaso et al., 2010). Forward and reverse reads were joined using fastqjoin and the data returned to the QIIME platform for operatational taxonomic unit (OTU) picking, alpha diversity, beta diversity, and further analysis. We applied a machine learning approach to identify taxa that discriminated between poised and unpoised electrode communities using the pamR package in R (Tibshirani et al., 2002), and applied a constrained correspondence analysis using the vegan package in R (Oksanen et al., 2013; R Core Team, 2013).

## Results and Discussion

### CH_4_ and N_2_/N_2_O Emissions

We measured CH_4_ emissions bi-weekly in each of 12 chambers split between two riparian zone sites over the 6-weeks experimental period and calculated the absolute average CH_4_ emission (**Table 1**). We observed differences between the two riparian zone sites, which were 50 m apart, in regards to CH_4_ emissions, showing that this specific riparian zone was heterogeneous. The absolute average CH_4_ emissions at the upstream site were considerably higher compared to the downstream site; absolute average CH_4_ emissions with the unpoised electrode (control) chambers were 1.96 ± 1.42 mg CH_4_^∗^m^-2∗^h^-1^ at the upstream site compared to 0.79 ± 0.65 mg CH_4_^∗^m^-2∗^h^-1^ at the downstream site (**Table 1**). On average, the absolute CH_4_ emissions from the poised electrode chambers at the upstream site were ∼50% lower than those from the unpoised electrode chambers, but due to the large temporal error not significantly different (*p* = 0.55, two-tailed *t*-test). At, the downstream site the absolute average CH_4_ emissions from the poised electrode chambers were similar compared to those from the unpoised electrode chambers (*p* = 0.91, two-tailed *t*-test; **Table 1**). However, there were large temporal trends throughout the experimental period. The CH_4_ emissions for each chamber were highest at the beginning of the experiment (earlier in the spring) and decreased throughout the 6-week experimental period (data not shown).

**Table 1:** Average CH_4_ emissions, N_2_/N_2_O emissions, SO_4_^2-^ concentrations, and Cl^-^ concentrations from chambers with poised and unpoised electrodes at both the upstream and downstream sites during the experimental period.

Function or environmental parameter	Upstream poised	Upstream unpoised	Downstream poised	Downstream unpoised
CH_4_ emission (mg CH_4_^∗^m^-2∗^h^-1^)	0.96 ± 0.86	1.96 ± 1.42	0.93 ± 1.1	0.79 ± 0.65
N_2_/N_2_O emission (μg N_2_O-N^∗^m^-2∗^h^-1^)	1793 ± 1794	2138 ± 2136	2394 ± 3287	2179 ± 2873
SO_4_^2-^ concentration (ppm)	15.1 ± 14.6	38.2 ± 31.2	41.4 ± 35.8	45.5 ± 32.8
Cl^-^ concentration (ppm)	88.5 ± 39.7	107 ± 41.2	103 ± 63.0	218 ± 142

*Large error is due to temporal changes during the experimental time period; as such, in further data analysis the groups were considered as paired samples on each measurement day (i.e., poised vs. unpoised) to remove the impacts of temporal trends when determining the effects of electrode-based manipulation. Error indicates standard error. *N* = 54 for all measurements.*

**FIGURE 1:**
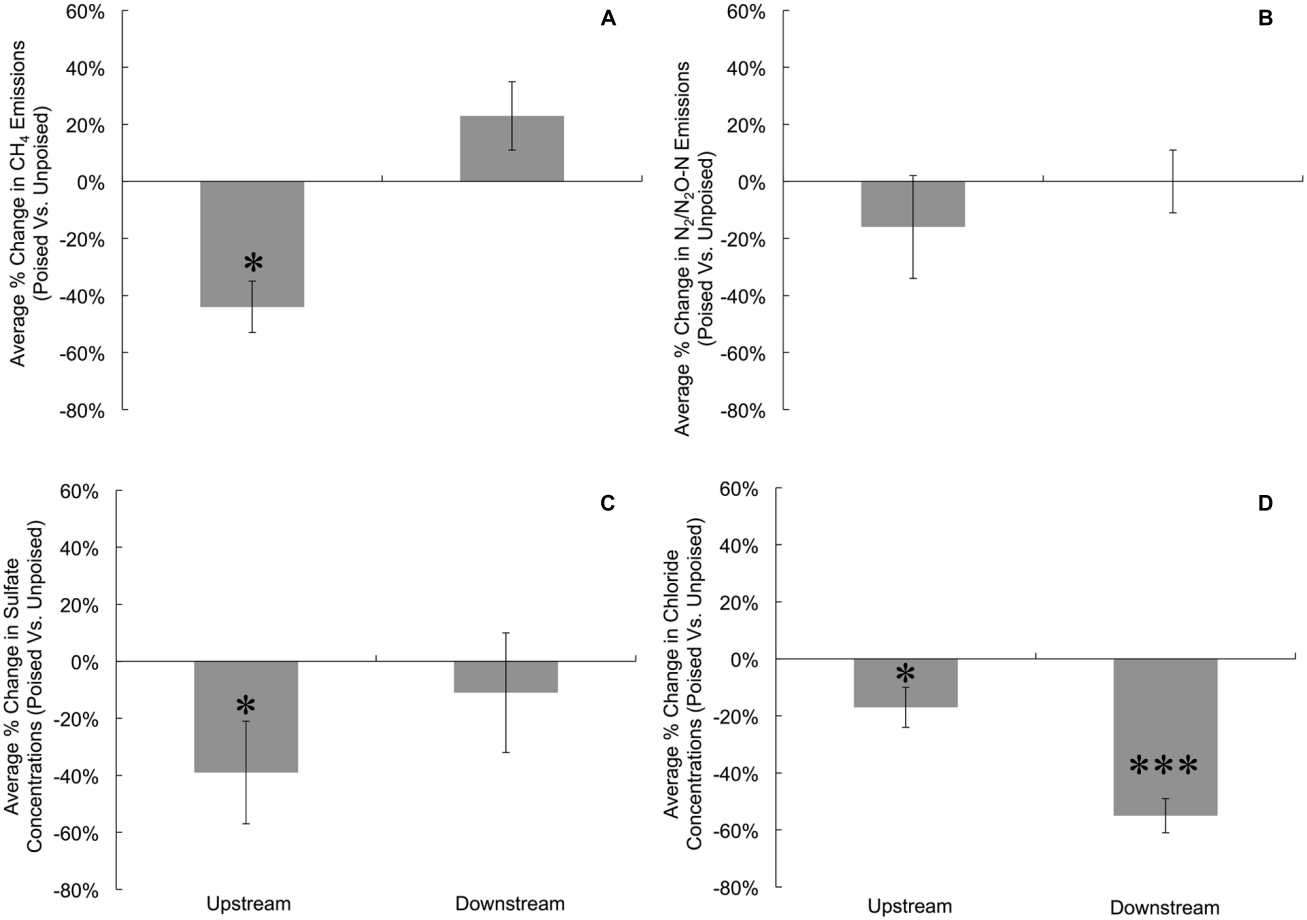
**Average percent change in gas emissions (poised:unpoised) for CH_4_**(A)** and N_2_/N_2_O **(B)**, as well as average percent change in the concentrations of SO_4_^2-^**(C)** and Cl^-^**(D)** for upstream and downstream sites during the duration of the experiment.** To account for temporal variations, gas emissions and anion concentrations from chambers with poised electrodes were compared against those with unpoised electrodes for each site on each measurement day (i.e., paired samples). Percent change indicates the change in chambers with poised electrodes compared against those with unpoised electrodes. Significant differences at are noted at the ^∗^*p* = 0.05 and ^∗∗∗^*p* = 0.001 thresholds from paired two-tailed student *t*-tests. Error bars indicate standard error.

To more precisely determine the effects of potentiostatic manipulation, we normalized average CH_4_ emissions (mg CH_4_^∗^m^-2∗^h^-1^) from chambers with poised electrodes against the CH_4_ emissions from chambers with unpoised electrodes at each site for each measurement day, resulting in an average percentage change in CH_4_ emission ratios of poised vs. unpoised electrode chambers (**Figure 1A**). At the upstream site, we observed a 44% reduction in normalized CH_4_ emission (*p* = 0.017, two-tailed paired *t*-test) for the poised vs. unpoised electrode chambers (**Figure 1A**). This is in agreement with our hypothesis: placement of iron(III)/humic substance-mimicking electrodes will reduce the emission rate methane compared to the control in a riparian zone, since it is thermodynamically more attractive to sustain iron/humic substance reduction (i.e., iron(III)/humic substances as the terminal electron acceptor) than methanogenesis (i.e., CO_2_ as the terminal electron acceptor). However, for the downstream site for which we observed lower soil temperatures (Supplementary Figure S6), lower pH (Supplementary Figure S7), more vegetation cover, and less direct sunlight, we did not observe a significantly average change in normalized CH_4_ emissions between the poised vs. unpoised chambers (*p* = 0.61, two-tailed paired *t*-test; **Figure 1A**). Noteworthy is that the temperature difference between the upstream and downstream sites may have caused for a small error in our data (∼1%). Based on our hypotheses, we anticipated lower CH_4_ emissions for the poised electrode chambers. It remains, however, unclear why the normalized CH_4_ emissions were indeed lower at the upstream site and not at the downstream site with poised electrodes. This could be due to environmental differences between sites (e.g., soil temperature, pH), but further work is required to determine the precise reason(s).

There were no differences in the absolute average N_2_/N_2_O emissions (μg N_2_O-N^∗^m^-2∗^h^-1^) between sites or treatments (**Table 1**). These data were also compared using percent change based on paired sample analysis in the same manner as the normalized CH_4_ emissions, and there were no significant changes between groups at either site (upstream: *p* = 0.051; downstream: *p* = 0.521, two-tailed paired *t*-test, **Figure 1B**). Regardless, the discrepancies with our CH_4_ emission results from adjacent field sites show the heterogeneous character of field research, and the importance to test results in the field rather than only at the bench.

### Environmental Parameters

We compared several environmental parameters that we measured for upstream and downstream sites during the study period. Soil temperature generally increased during the experimental period (and thus throughout the spring season), and ranged between 6.8 and 19.1°C (Supplementary Figure S6). On a daily basis throughout the 6-week period, the average soil temperatures at the upstream site were 1.5°C higher than that of the downstream site (*p* < 0.001, two-tailed *t*-test). The pH values across both sites were close to each other and near neutral, with the upstream site averaging at 7.3 ± 0.2 and the downstream site at 7.2 ± 0.2 (Supplementary Figure S7). There were no differences in soil electrical conductivity between sites or control and treatment collars (Supplementary Figure S8); the average soil conductivity across both sites was 403 ± 165 μS/cm.

Dissolved oxygen was low across both sites due to water tables at or near the soil surface (for more than 75% of the experimental period), and both sites showed average concentrations of less than 1 mg L^-1^ dissolved oxygen (Supplementary Figure S9). Both NO_3_^-^ and NO_2_^-^ were low (<1 ppm) across sites and treatments, and were below detection limit (0.1 ppm) for most samples (consistent with the relatively low N_2_/N_2_O emissions rates). We did not observe a difference between sites and treatments for total iron concentrations from soil pore water, which averaged 10.97 ± 9.0 μM. Approximately 95% of this iron was in the reduced form (Fe^2+^), and there were no differences between sites or treatments in iron speciation. At the upstream site, SO_4_^2-^ concentrations were lower in chambers with poised electrodes than those with unpoised electrodes, however, there was not a significant difference in absolute SO_4_^2-^ concentrations at the downstream site (**Table 1**). These values were corrected in the same manner as the normalized CH_4_ emission rates, visualizing an average reduction in normalized SO_4_^2-^ concentration of 39% with poised vs. unpoised electrodes (*p* = 0.02, two-tailed paired *t*-test; **Figure 1C**). It is unclear whether the difference in SO_4_^2-^ concentrations at the upstream site was related to the reduction in CH_4_ emissions, although sulfate reduction is more energetically favorable than methanogenesis, but less favorable than iron reduction (Regnier et al., 2011). Based on energetics, we would have anticipated a higher concentration of sulfate (less sulfate reduction) with a poised electrode. A conclusive explanation is elusive, but we can speculate: in some cases, microbial iron-reducing bacteria have been found to coincide with sulfate-reducing microbes (*Desulfovibrio* sp.; Lentini et al., 2012), and it is possible that a synergistic relationship between iron- and sulfate-reducers is responsible for the decreased sulfate concentrations in the chambers with poised electrodes at the upstream site. This co-occurrence is supported by the simultaneous normalized CH_4_ emission reduction (via mimicked iron reduction with electrodes) and decreased normalized SO_4_^2-^ concentration (via increased sulfate reduction) at the upstream site, while neither decrease occurred at the downstream site.

Absolute Cl^-^ concentrations were lower in pore water collected from chambers containing poised electrodes at both sampling sites (**Table 1**); these values were corrected in the same manner as normalized CH_4_ emission rates. At the upstream sites, chambers with poised electrodes had normalized Cl^-^ concentrations that were 17% lower than those with unpoised electrodes (*p* = 0.007, two-tailed paired *t*-test); at the downstream site, normalized Cl^-^ concentrations were, on average, 55% lower than in chambers with unpoised electrodes (*p* < 0.0005, two-tailed paired *t*-test; **Figure 1D**). These lower Cl^-^ concentrations were a result of Cl^-^ transport from the inside to the outside chamber through the anion exchange membrane because of ion imbalances due to the electrochemical activity (Rozendal et al., 2006; Fornero et al., 2010b).

### Microbial Community Dynamics

**FIGURE 2:**
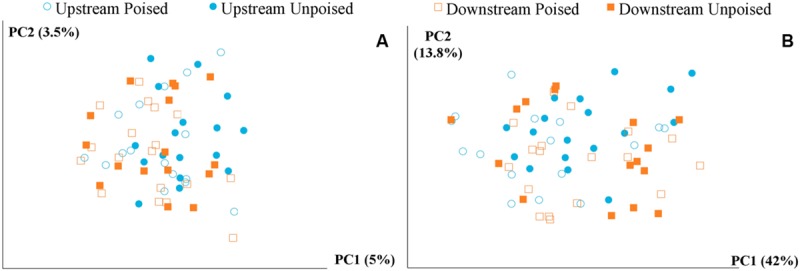
**Principal coordinate analysis of the beta diversity of microbial communities shown using unweighted **(A)** and weighted **(B)** UniFrac distances.** The weighted **(B)** method explains more variation (55.8%) in the first two principal coordinates than the unweighted **(A)** method (8.5%); however, in both cases there are no clear clusters differentiating between sites (upstream or downstream) or treatment (poised or unpoised electrodes).

**FIGURE 3:**
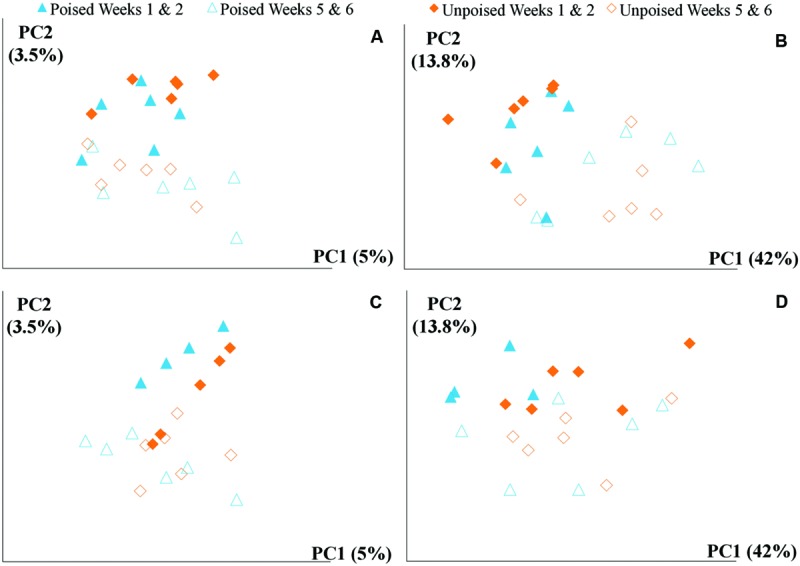
**Principal coordinate analysis of beta diversity at upstream and the downstream sites using unweighted and weighted UniFrac distances: **(A)** upstream site, unweighted UniFrac; **(B)** upstream site, weighted UniFrac; **(C)** downstream site, unweighted UniFrac; and **(D)** downstream site, weighted UniFrac.** The weighted **(B,D)** method explains more variation (55.8%) in the first two principal coordinates than the unweighted **(A,B)** method (8.5%); however, in both cases there are no clear clusters differentiating between sites (upstream or downstream) or treatment (poised or unpoised electrodes). There was some clustering between time points (weeks 1 and 2 or weeks 5 and 6), suggesting that shifts in community were primarily driven by environmental conditions rather than manipulation.

We obtained a total of 72 samples (69 of which were sequenced; 3 samples had too low of a DNA yield to be sequenced) from the 12 chambers during 6 weeks and achieved an average sequencing coverage of 29,782 sequences (16S rRNA gene) per sample with an average length of 251 bp. We first analyzed the beta diversity, which quantifies the differentiation between sample communities, using UniFrac distances (Lozupone and Knight, 2005). Based on the differences in CH_4_ emissions and environmental parameters, we had expected microbial communities to be similar to each other if they came from the same site or treatment. However, principal coordinate analysis of both unweighted UniFrac distances and weighted UniFrac distances (weighted takes into account relative abundances of OTUs within samples) did not reveal any clustering of samples by site or treatment (**Figures 2A,B**). However, there was a clear clustering of samples with time, which was highlighted by UniFrac distances between the first 2 weeks (weeks 1–2) and last 2 weeks (weeks 5–6) of the experimental period (**Figure 3**). This result likely suggests that most of the broad differences in the microbial community phylogenetic structure were based simply on changing environmental parameters and community function with time, rather than experimental treatment.

**FIGURE 4:**
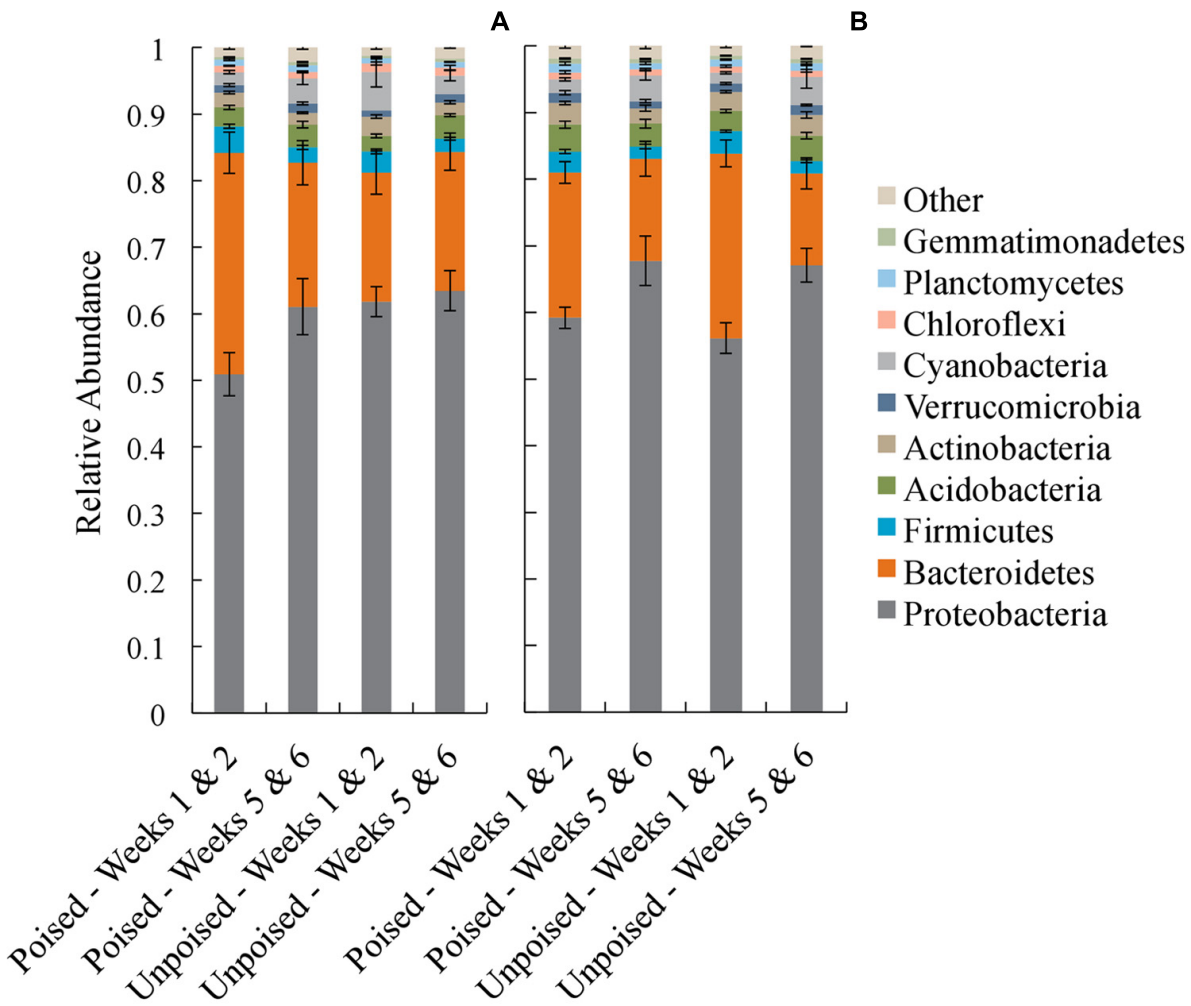
**Average taxonomic summaries (phylum level) of the microbial communities from the upstream **(A)** and downstream **(B)** sites.** Communities are grouped in the first 2 weeks (weeks 1–2) and the last 2 weeks (weeks 5–6). Error bars indicate standard error.

In each of the electrode samples, over 80% of the 16S rRNA gene sequences were classified in the phyla Proteobacteria and Bacteroidetes, with a remaining 1–6% of sequences falling in the phyla Firmicutes, Acidobacteria, Actinobacteria, Verrucomicrobia, Cyanobacteria, Chloroflexi, Planctomycetes, and Gemmatimonadetes (**Figure 4**). Similar to the beta diversity data analysis, we grouped the taxonomy data for weeks 1–2 and weeks 5–6 with the objective to identify phyla that changed during the experimental period of 6 weeks. At the upstream site, the community composition at the poised electrodes between weeks 1–2 and 5–6 showed a 10% increase in Proteobacteria (*p* = 0.19, two-tailed *t*-test) and a 35% decrease in Bacteroidetes (*p* = 0.04, two-tailed *t*-test) during the experimental period. Proteobacteria and Bacteroidetes populations were not different at the unpoised electrodes between weeks 1–2 and 5–6 (**Figure 4A**). At the downstream site, both poised and unpoised electrodes enriched for Proteobacteria between weeks 1–2 and 5–6, which was accompanied by a decrease in relative abundance of Bacteroidetes (**Figure 4B**). Specifically, the poised electrodes resulted in a 14% increase in Proteobacteria and a 30% decrease in Bacteroidetes (*p* = 0.06 for both phyla, two-tailed *t*-test) while the unpoised electrodes caused a 20% increase in Proteobacteria and a 50% decrease in Bacteroidetes (*p* < 0.01 for both phyla, two-tailed *t*-test) between weeks 1–2 and 5–6. The increase in Proteobacteria in both poised and unpoised treatments could be due to the presence of the graphite electrodes, which provide a conductive surface that promotes both biofilm growth and long-range electron transport through soil microenvironments (even when unpoised; Erable et al., 2011; Kato et al., 2012; Friedman et al., 2013). Thus, with this phyla richness analysis we found that three out of four comparisons of week 1–2 and week 5–6 binned data showed a similar trend of increasing Proteobacteria, but this included the poised *and* unpoised electrodes at one site.

**FIGURE 5:**
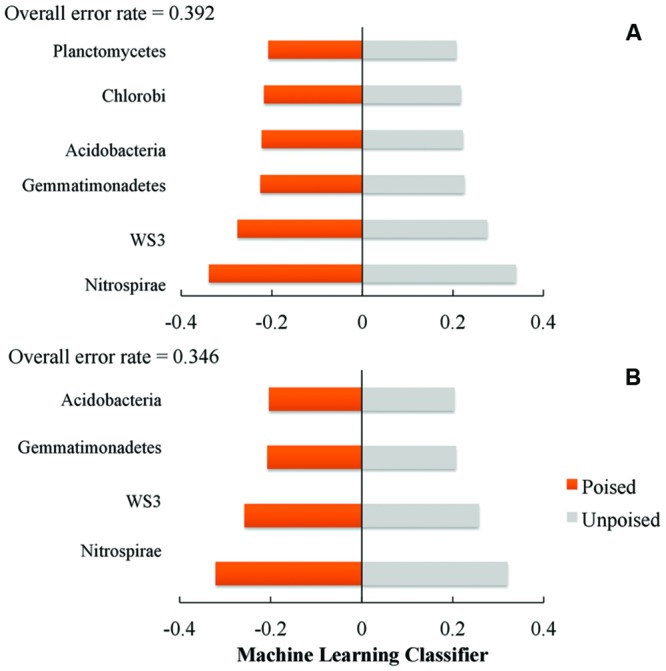
**Machine learning analysis using phylum level taxonomy shows the phyla that are most indicative of poised or unpoised electrode communities across: **(A)** both sites; and **(B)** just the upstream site.** Machine learning classifier is a nearest shrunken centroid classifier calculated using the pamR package in R.

We used a machine learning approach to find OTUs that are predictive of samples taken from either poised or unpoised electrodes (**Figure 5**). We applied this to OTU abundance profiles from all samples (**Figure 5A**) as well as data from only the upstream site samples (**Figure 5B**). Upon cross-validation, the machine-learning algorithm returned a relatively high error rate (0.392 and 0.346 for both sites, and the upstream site, respectively), indicating that there were no indicator OTUs that could differentiate poised from unpoised electrode conditions. This machine learning approach confirmed our other findings from the principal coordinate analysis of UniFrac-based beta diversity (**Figures 2** and **3**) and the other taxonomic classification analysis (**Figure 4**) that the bioelectrochemical manipulation did not cause systematic, or reproducible, changes in the microbial membership.

### Linking Microbial Community Dynamics to Gas Emissions and Environmental Parameters

**FIGURE 6:**
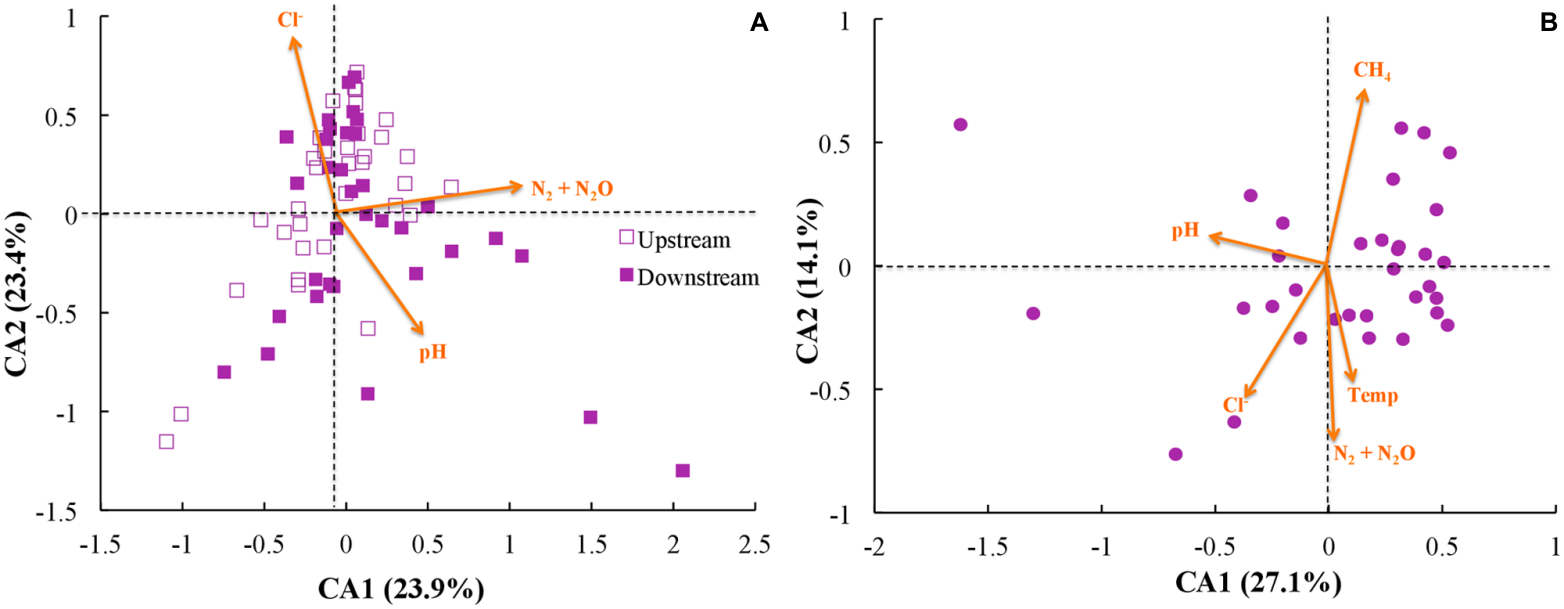
**Correspondence analysis (CA) using rarified and filtered OTU tables, gas emissions, and environmental metadata for: **(A)** all samples; and **(B)** only samples from the upstream site.** Points indicate samples while arrows indicate vectors for both gas emissions and environmental metadata, with longer arrows indicating higher influence for those parameters.

Next, we investigated whether community function (e.g., gas emission) or environmental parameters were associated with microbial community membership. We performed correspondence analysis to compare OTU abundance profiles with gas emission and environmental metadata (rarified with 500 sequences per sample; **Figure 6**). First, we performed principal component analysis with our OTU tables, and second we performed correspondence analysis to map environmental metadata onto the principal component results as vectors. The resulting direction of the vector (arrow) indicates that the specific variable corresponded strongly with community structure, while the resulting length of the vector indicates the magnitude of the association (i.e., longer arrows indicate a more significant weighting of the given variable in the overall ordination). When we used data from across all sites and treatments, the variables of strongest correspondence with the OTU tables were: N_2_/N_2_O emissions, pH, and Cl^-^ concentrations (**Figure 6A**). When limiting the analysis to the upstream site (where unpoised electrode chambers had higher CH_4_ emissions), however, the CH_4_ emissions were the most strongly weighted variable, with N_2_/N_2_O emissions, pH, Cl^-^ concentrations, and temperature being other variables (**Figure 6B**). Noteworthy is that temperature and time as a vector cannot be distinguished in our work because of slowly rising soil temperatures during our experimental period (Supplementary Figure S6).

### Future Work Critical to Elucidate Microbial Community Dynamics in Riparian Zones

Riparian zones have the capability to buffer aquatic ecosystems from the adjacent land and are important ecosystems for watershed management, especially in agricultural areas (McClain et al., 2003; Burt et al., 2010). Many studies have attempted to directly link hydrologic parameters (e.g., water table depth, groundwater flow patterns) to both denitrification rates and greenhouse gas emissions (Vidon and Hill, 2004; Aronson et al., 2012; Audet et al., 2013; McPhillips et al., 2015). However, microbial communities are the drivers of subsurface biogeochemical transformations, and as such it is critical to quantitatively link microbial community dynamics to ecosystem level parameters and functions (Raes and Bork, 2008; Burt et al., 2010; Morales and Holben, 2011). There are often chemical or biological links (or both) between different ecosystem processes, such as temperature, groundwater flow, plant growth, and denitrification (Teh et al., 2008; Liptzin and Silver, 2009; Dubinsky et al., 2010; Vidon et al., 2010; Frei et al., 2012).

When we manipulated the soil redox environment using poised electrodes mimicking iron(III), there was a significant decrease in CH_4_ emissions from the upstream site (**Figure 1A**), which we anticipated because the presence of a terminal electron acceptor with the redox potential of iron(III) for iron reduction is thermodynamically more favorable compared to carbon dioxide for methanogenesis. Furthermore, manipulation resulted in a decrease in SO_4_^2-^ and Cl^-^ concentrations in soil porewater (**Figures 1C,D**). However, at the downstream site, which had a lower magnitude of emissions (nearly 50%) from the chambers with unpoised electrodes (i.e., controls), poised electrodes had no effect on CH_4_ emissions. This is likely due to the lower overall CH_4_ emission rates at this site, which would preclude inhibition via bioelectrochemical manipulation since methanogenic activity was already low.

We also observed, together with the reduction in CH_4_ emission from the upstream site, an increase in the relative abundance of Proteobacteria and a decrease in Bacteroidetes (**Figure 4A**). However, we found no systematic link between community structure and experimental treatment (poised vs. unpoised electrodes; **Figures 2** and **3**). Further quantitative analysis showed that these microbial community changes corresponded with differences in the environmental parameters (pH and Cl^-^ concentrations) and CH_4_ and N_2_/N_2_O emissions (**Figure 6**).

The indirect alteration of microbial community structure and suppression of CH_4_ emissions at the upstream site in conjunction with bioelectrochemical manipulation (using soil-based electrodes) demonstrates the fragile balance that governs biogeochemical cycles in these soils, and highlights the measurable impact that microbial competition has on ecosystem-scale processes. It is clear that microbial community structure and function is subject to influence from a wide array of biological, chemical, and physical factors, which in turn can have a measurable impact on the landscape level. Accurate modeling of biogeochemical processes is important for predicting responses to climate change, determining regulatory limits for anthropogenic pollutants, and designing effective best management practices (Vidon et al., 2010; Meng et al., 2011; Riley et al., 2011). As such, a deeper comprehension of subsurface microbial ecosystems, their responses to environmental conditions across spatial and temporal gradients, and their impacts on larger-scale function, is critical for improving model accuracy, and further studies are certainly warranted.

In summary, we manipulated the sediment redox environment by poising electrodes capable of being used as electron acceptors for iron(III)-reducing microbes and observed a response in ecosystem function (in this case, CH_4_ emissions) at one riparian zone site. Poised electrodes provided an inexhaustible source of electron acceptor for iron reducers, and, at our upstream riparian zone site, resulted in reduced CH_4_ emissions that could be a result of iron- or humic substance-reducers outcompeting methanogens for carbon sources and nutrients. However, we did not see such changes in CH_4_ emissions at the downstream site. Furthermore, we showed that bioelectrochemical manipulation had minimal effects on the microbial community structure. This was confirmed by machine learning analysis, which was unable to develop an algorithm to predict sample grouping with an error rate below 30%. We did, however, find a correspondence between the community composition and the function of the microbiota (CH_4_ emissions). Despite the lack of direct changes in microbial community structure at the upstream site with poised electrodes, the reduction (∼45%) of CH_4_ emissions together with its correspondence to the composition of microbiota suggests that the balance between competing anaerobic microbial processes can have a major impact on landscape-level processes.

Key Points:

• Potentiostatic manipulation was used to change redox dynamics in riparian soils.
• Manipulation inhibited CH_4_ emissions but did not change the microbial community.
• Microbial community dynamics corresponded to environment and ecosystem function.

## Author Contributions

EF, LM, MW, and LA designed the study; EF and LM performed the field research; EF, LM, and AP performed the sample analysis; EF, LM, JW, AP, RL, MW, and LA analyzed the data, EF, LM, JW, AP, RL, MW, and LA wrote the manuscript.

## Conflict of Interest Statement

The authors declare that the research was conducted in the absence of any commercial or financial relationships that could be construed as a potential conflict of interest.
