# Supplementary material for: Methane Emission in a Specific Riparian-Zone Sediment Decreased with Bioelectrochemical Manipulation and Corresponded to the Microbial Community Dynamics
Source: Front Microbiol. 2016 Jan 11;6:1523. doi: 10.3389/fmicb.2015.01523 (PMC4707442; doi:10.3389/fmicb.2015.01523)
Supplement: Supplementary file 1 [file Data_Sheet_1.DOCX]

**Figure S1.** Schematic of the chamber and electrode configuration used in this experiment.


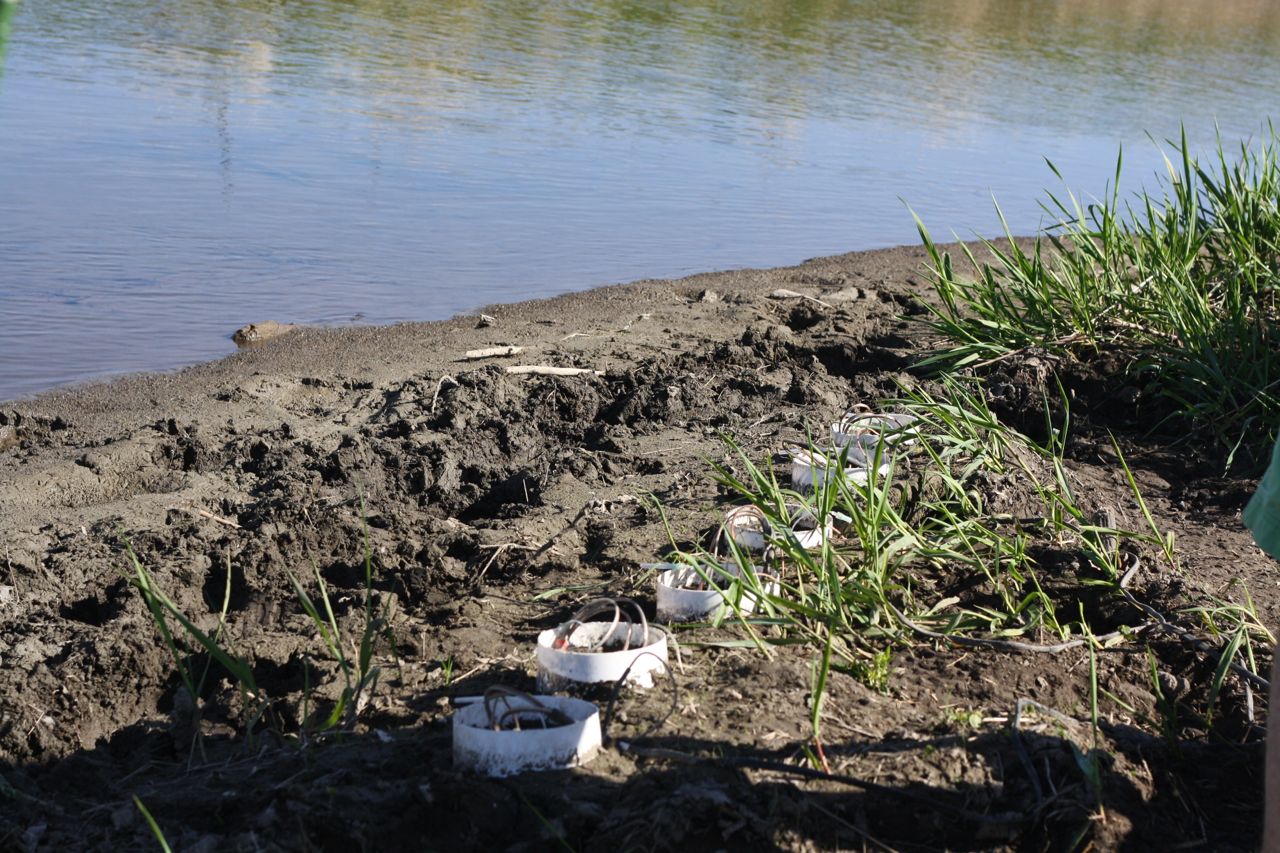


**Figure S2.** View of the six experimental collars at the upstream site.


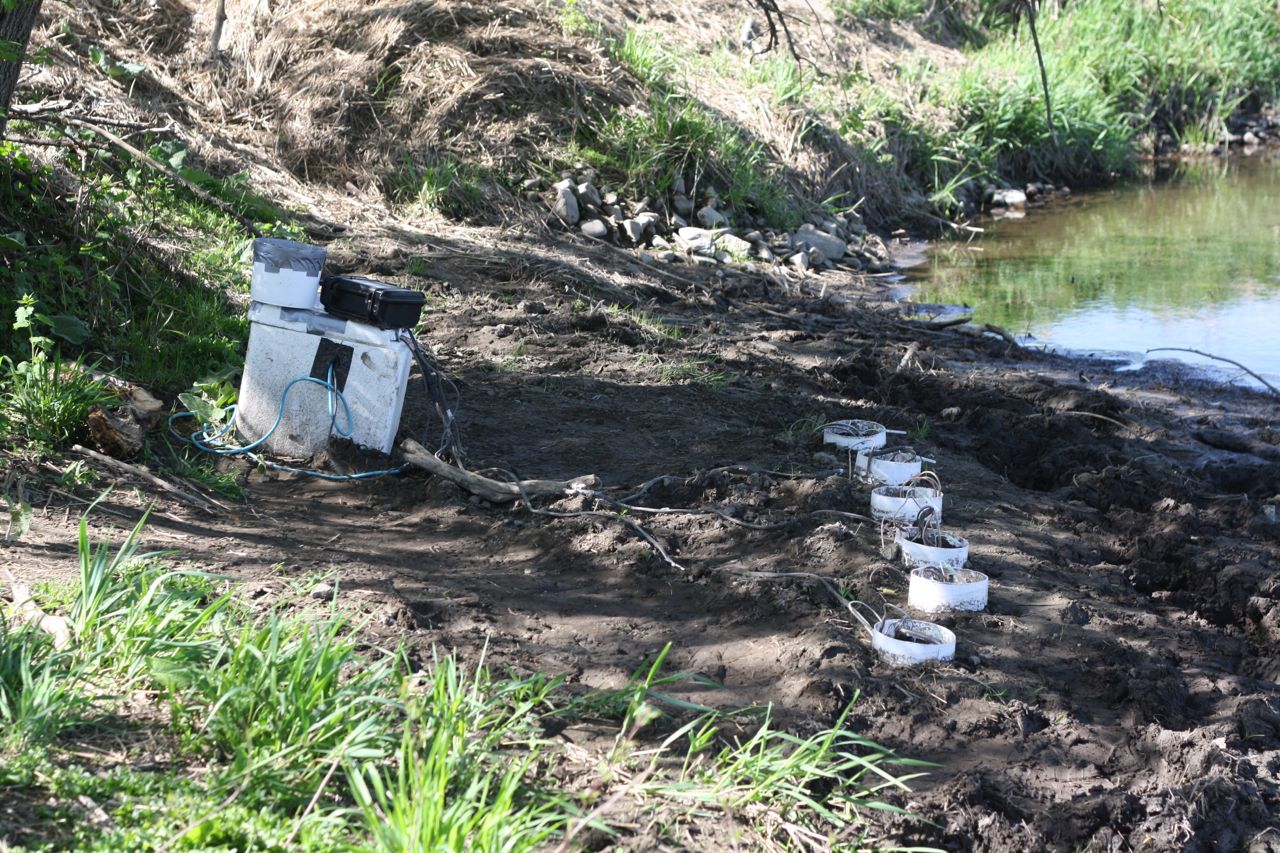


**Figure S3.** View of the six experimental collars plus the battery box and potentiostat at the downstream site.

**Figure S4.** Raw electric current data for the upstream poised electrodes.

**Figure S5.** Raw electric current data for the downstream poised electrodes.

**
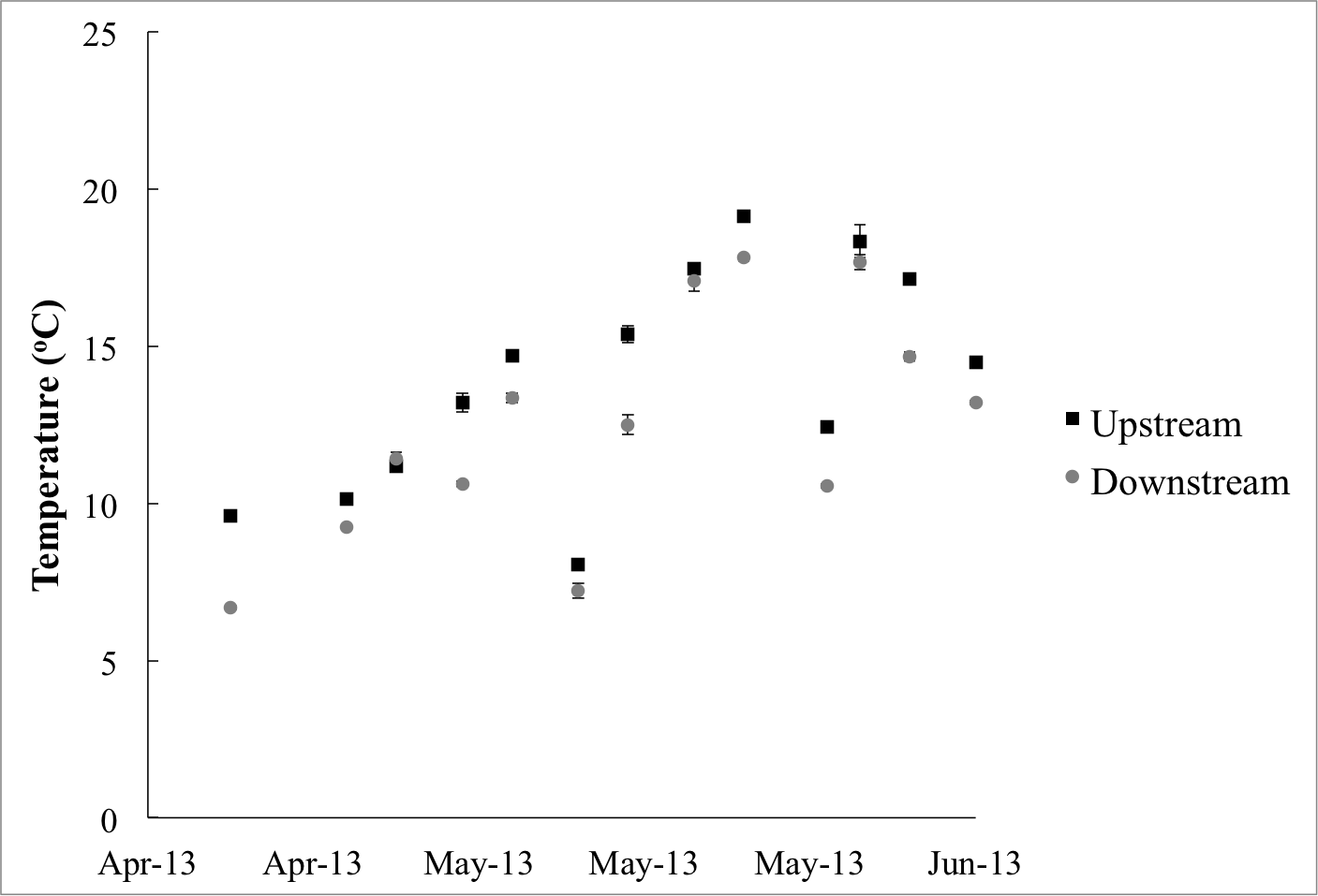
**

**Figure S6.** Soil temperature measurements from the upstream and downstream sites over the course of the experimental period. Each point is the average of all six chambers (three with poised electrodes and three with unpoised electrodes) for that day, as there were no differences between chambers with poised and unpoised electrodes. Error bars show standard error.

**Figure S7.** Soil pH measurements from the upstream and downstream sites over the course of the experimental period. Each point is the average of all six chambers (three with poised electrodes and three with unpoised electrodes) for that day, as there were no differences between chambers with poised and unpoised electrodes. Error bars show standard error.

**
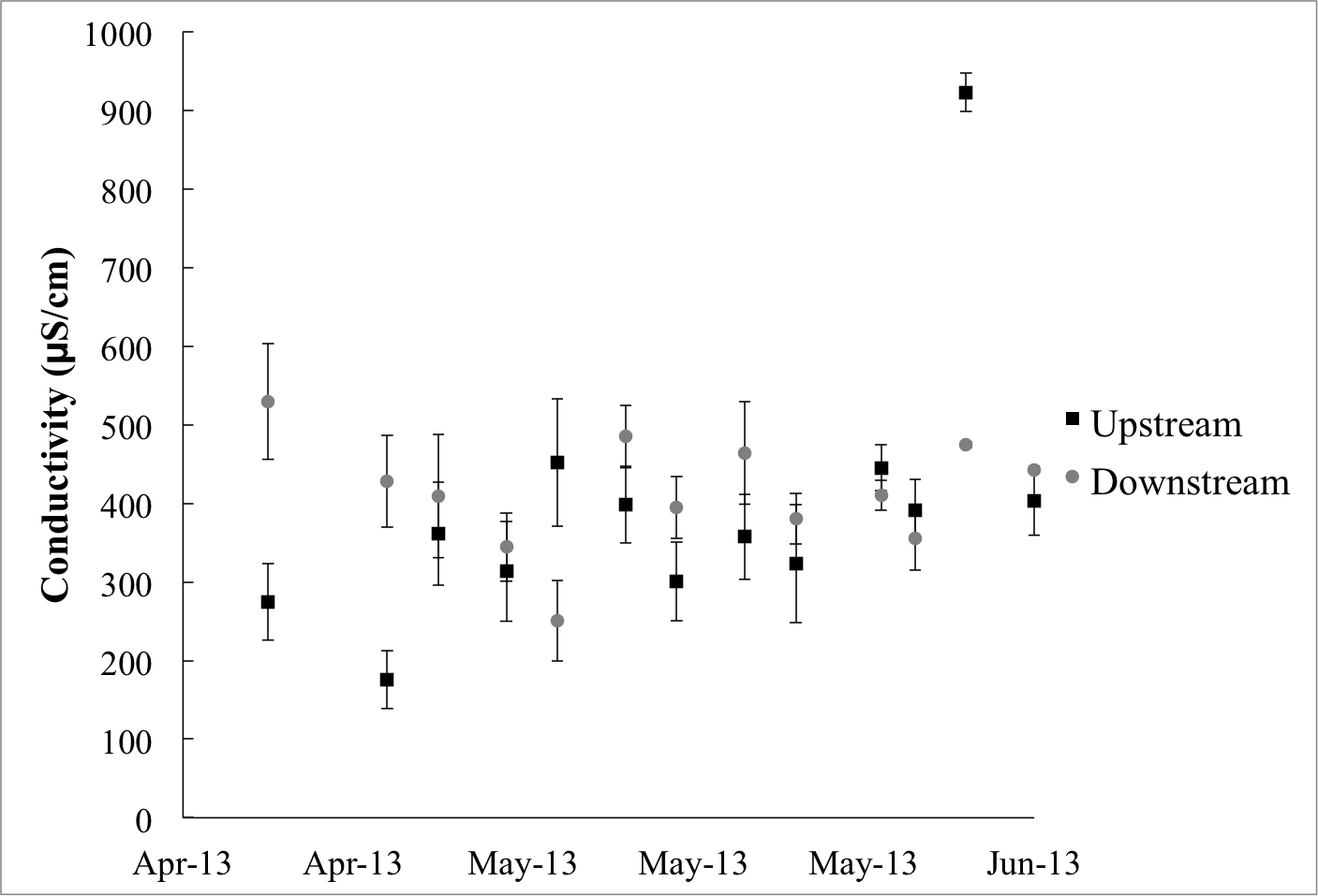
**

**Figure S8.** Soil conductivity measurements from the upstream and downstream sites over the course of the experimental period. Each point is the average of all six chambers (three with poised electrodes and three with unpoised electrodes) for that day, as there were no differences between chambers with poised and unpoised electrodes. Error bars show standard error.

**
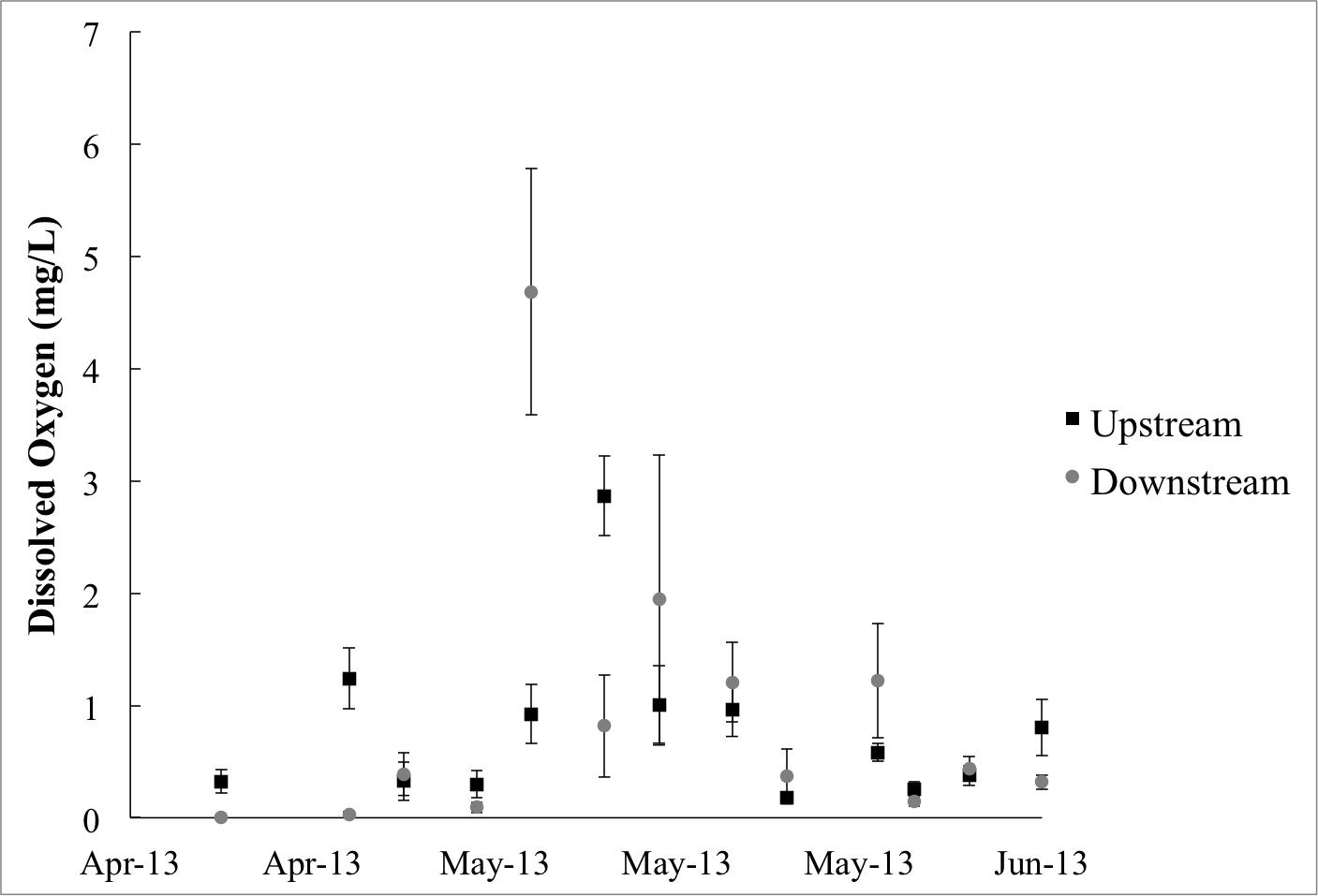
**

**Figure S9.** Dissolved oxygen measurements from the upstream and downstream sites over the course of the experimental period. Each point is the average of all six chambers (three with poised electrodes and three with unpoised electrodes) for that day, as there were no differences between chambers with poised and unpoised electrodes. Error bars show standard error.
